# Mannose-Modified Chitosan-Nanoparticle-Based *Salmonella* Subunit OralVaccine-Induced Immune Response and Efficacy in a Challenge Trial in Broilers

**DOI:** 10.3390/vaccines8020299

**Published:** 2020-06-11

**Authors:** Yi Han, Sankar Renu, Veerupaxagouda Patil, Jennifer Schrock, Ninoshkaly Feliciano-Ruiz, Ramesh Selvaraj, Gourapura J. Renukaradhya

**Affiliations:** 1 Food Animal Health Research Program, Ohio Agricultural Research and Development Center, 1680 Madison Avenue, Wooster, OH 44691, USA; han.1201@buckeyemail.osu.edu (Y.H.); renu.2@osu.edu (S.R.); patil.202@osu.edu (V.P.); schrock.57@osu.edu (J.S.); feliciano-ruiz.1@buckeyemail.osu.edu (N.F.-R.); 2 Department of Veterinary Preventive Medicine, College of Veterinary Medicine, The Ohio State University, Columbus, OH 43210, USA; 3 Department of Poultry Science, University of Georgia, Athens, GA 30602, USA; selvaraj@uga.edu

**Keywords:** broilers, *Salmonella* Enteritidis, chitosan nanoparticle, mannose modification, antibody response, innate immunity, cell-mediated immunity

## Abstract

Controlling *Salmonella enterica* serovar Enteritidis (SE) infection in broilers is a huge challenge. In this study, our objective was to improve the efficacy of a chitosan nanoparticle (CS)-based *Salmonella* subunit vaccine for SE, containing immunogenic outer membrane proteins (OMP) and flagellin (FLA), called the CS(OMP+FLA) vaccine, by surface conjugating it with mannose to target dendritic cells, and comparing the immune responses and efficacy with a commercial live *Salmonella* vaccine in broilers. The CS(OMP+FLA)-based vaccines were administered orally at age 3 days and as a booster dose after three weeks, and the broilers were challenged with SE at 5 weeks of age. Birds were sacrificed 10 days post-challenge and it was observed that CS(OMP+FLA) vaccine surface conjugated with both mannose and FLA produced the greatest SE reduction, by over 1 log_10_ colony forming unit per gram of the cecal content, which was comparable to a commercial live vaccine. Immunologically, specific mucosal antibody responses were enhanced by FLA-surface-coated CS(OMP+FLA) vaccine, and mannose-bound CS(OMP+FLA) improved the cellular immune response. In addition, increased mRNA expression of Toll-like receptors and cytokine was observed in CS(OMP+FLA)-based-vaccinated birds. The commercial live vaccine failed to induce any such substantial immune response, except that they had a slightly improved T helper cell frequency. Our data suggest that FLA-coated and mannose-modified CS(OMP+FLA) vaccine induced robust innate and adaptive cell-mediated immune responses and substantially reduced the *Salmonella* load in the intestines of broilers.

## 1. Introduction

*Salmonella enterica* serovar Enteritidis (SE) is a Gram-negative bacterium that causes the majority of foodborne illness associated with broilers and is responsible for major economic losses to the U.S poultry industry [1]. Approximately 9% of samples from poultry production are positive for *Salmonella* [2]. *Salmonella* contamination accounted for the greatest number of FDA-regulated food recalls during 2003 through 2011 [3]. Through an effective vaccination approach, Salmonellosis in humans can be substantially decreased by reducing *Salmonella* colonization in poultry. Unfortunately, there are limited commercially available *Salmonella* vaccines for use in broilers, and none of them provides protective immunity until slaughter. Commercial live *Salmonella* vaccines are unsafe as the live vaccine strains (attenuated by natural selection or genetic engineering) are potentially released into the environment and contaminate the human food chain [4]. Currently, due to a lack of effective vaccines and safety reasons, less than 1% of broilers receive a live *Salmonella* spray vaccine once [5], and FDA regulations prohibit its use within 21 days of slaughter.

Consumption of poultry meat contaminated with *Salmonella* is an important cause of infections in humans. Therefore, there is a pressing demand for development of novel control methods that protect broilers from the day of hatch until slaughter against *Salmonella* infection. Our previous vaccine trial in broilers inoculated orally with chitosan nanoparticles (CS) entrapped with SE outer membrane proteins (OMP) and flagellin (FLA) and surface-coated with FLA, called the CS(OMP+FLA)-F vaccine, was shown to reduce the challenge SE load by 0.7 log_10_ CFU/g in the cecal content [5]. This outcome was associated with the secretion of increased antigen-specific mucosal and systemic antibodies, splenocytes proliferation, and the frequency of IFNγ-producing T-cell responses. A similar study in layer chickens with CS(OMP+FLA)-F vaccine delivered orally targeted intestinal immune sites and induced mucosal antibody and cell-mediated immune responses, resulting in reduced challenge SE load [6]. Additionally, CS(OMP+FLA)-F-vaccine-treated chicken immune cells showed enhancement of various Toll-like receptors (TLRs) and Th1 and Th2 cytokine gene expression [6].

Mannose-ligand-binding C-type mannose receptor is mainly expressed in the dendritic cells (DCs) and macrophages [7]. In an earlier study, mannose-ligand-modified CS carrying vaccine cargo administered orally was found to target and deliver the loaded antigen to gut DCs in mice [8]. Protein-antigen-encapsulated mannosylated chitosan microspheres delivered orally were shown to bind with mannose receptors on macrophages and induce mucosal antibody responses in mice [9]. Mannose-conjugated nanoparticles further improves its adjuvant effect [10], resulting in heightened immunity in the intestines of mice [11]. Therefore, in our present study, to improve the efficacy of the CS(OMP+FLA)-F vaccine, we conjugated mannose with or without FLA on the surface, CS(OMP+FLA)-F&M, and CS(OMP+FLA)-M formulations. These vaccine candidates were administered orally to broiler birds and evaluated for induced immune responses and efficacy compared to an orally delivered commercial live *Salmonella* vaccine (Poulvac^®^ ST). The Poulvac^®^ ST is a genetically modified *Salmonella* typhimurium (ST) strain, modified by deleting the aroA gene, and provides cross-protection against *Salmonella* Kentucky, Enteritidis, Heidelberg and Hadar in birds [12].

## 2. Material and Methods

### 2.1. Experimental Animals, Bacteria, and Vaccines Formulation

Day-old Cornish Cross breed broilers were purchased from a commercial hatchery (Ashland, OH, USA). Birds were confirmed *Salmonella*-free upon arrival by plating the cloacal swab samples on Xylose Lysine Deoxycholate and Brilliant Green agar plates (XLD; BGA, Sigma-Aldrich, St Louis, MO, USA). Chickens were reared on flooring with pine shavings as litter in an environmentally controlled BSL2 animal facility, and lighting was provided for 18 h/day. Birds were fed with a mash corn–soybean diet free from antibiotics. Feed and water were provided *ad libitum*. All animal handling procedures followed were as per the standards of the Institutional Laboratory Animal Care and Use Committee and Ethics for Animal Experiments at Ohio State University (Protocol number: 2016A00000060-R1).

The poultry isolate of SE, bacteriophage type 13A [13], was originally obtained from the USDA National Veterinary Services Laboratory (Ames, IA, USA).

Outer membrane proteins (OMP) and flagellin proteins (FLA) were isolated as previously described [14]. The vaccine formulations were prepared by an ionic gelation method and characterized as previously described [6,15]. The mannose-modified CS(OMP+FLA)-M and CS(OMP+FLA)-F&M vaccines were prepared as reported earlier [16,17], with few modifications. Briefly, for conjugation of mannose on chitosan, 1% (*w/v*) chitosan (Sigma, St Louis, MO, USA) solution was prepared in an aqueous solution of 1% acetic acid under magnetic stirring and pH adjusted to 4.3. Forty mg each of mannose and sodium triacetoxyborohydride mixture in 0.2 mL borate buffer was slowly added into 200 mg chitosan under magnetic stirring for 72 h at 56 °C. The mannose-conjugated chitosan was dialyzed against milli-Q-water for 48 h, lyophilized and stored until use. Ten mg mannose-conjugated chitosan was added into milli-Q-water (1 mg/mL) under magnetic stirring and incubated with 0.5 mg each of OMP and FLA protein in 1 mL 10 mM MOPS pH 7.4 buffer. Followed by the crosslinker sodium tripolyphosphate TPP (Sigma, St Louis, MO, USA), 2.5 mg in 5 mL (0.5 mg/mL) in milli-Q-water was added drop-by-drop using an insulin syringe. After 10 min of incubation, 150 µg FLA protein in 1 mL MOPS buffer was added to the formulation and the CS(OMP+FLA)-F&M was collected as a pellet after 10,976× *g* for 30 min centrifugation, suspended in milli-Q-water and used for vaccination. The CS (OMP+FLA)-F and CS (OMP+FLA)-M vaccines were prepared similarly but without mannose or FLA. In each dose of vaccines, an equal amount (5 μg each) of OMP and FLA were entrapped.

### 2.2. Experimental Design

On the day of hatch, 65 *Salmonella*-free day-old broilers were received from a commercial hatchery. Birds were randomly grouped into 7 groups. Treatment groups of chicks received 10 μg OMP+FLA antigens entrapped in three different vaccines CS(OMP+FLA)-F, CS(OMP+FLA)-M and CS(OMP+FLA)-F&M formulation beginning at 3 days of age (Table 1). The control soluble antigen group [10 μg antigens/bird, Sol.Ag (OMP+FLA)] was included. Another control group received a commercial live vaccine (PoulVac^®^ ST, Zoetis, Kalamazoo, MI, USA), inoculated twice as per the manufacturer’s instructions (1st dose sprayed on birds at age 3 days, and 2nd dose given through drinking water at 3 weeks). All groups received 2 doses of vaccine, and at 5 weeks of age blood and cloacal swab samples were collected. Except for the mock group, the remaining groups of birds were challenged orally with a pre-titrated dose of SE 5 × 10^8^ CFU/bird. All birds were euthanized at 10 days post-challenge, and samples of blood, cloacal swab, bile, small intestine, spleen, cecal tonsils and cecal content were collected. The aliquots of serum, cloacal swab fluid, small intestine wash fluid and bile samples were stored at −20 °C until analyzed for antibody response. Spleen was collected for use in splenocyte proliferation assay and for determining the frequency of specific T lymphocyte subsets (total T lymphocytes, T-helper lymphocytes, cytotoxic T lymphocytes and IFNγ-producing T lymphocytes) by flow cytometry. Bacterial shedding results were determined by plating cecal content (diluted with PBS according to the weight) on nalidixic-acid-resistant XLD agar and BGA plates.

### 2.3. Enzyme-Linked Immunosorbent Assay (ELISA)

The procedure for SE-specific antibody detection was performed as described previously [14]. Briefly, 96-well plates (Greiner bio-one, Frickenhausen, Germany) were coated with pre-titrated optimal amounts of OMP or FLA proteins (375 ng/well for IgA detection; 50 ng/well for IgG detection) in carbonate–bicarbonate buffer (pH 9.6), incubated for overnight at 4 °C and blocked with 5% (*v*/*v*) skim milk (Nestle, Vevey, Switzerland) diluted in PBS Tween-20 (0.05%) (PBS-T) for 1 h at 37 °C. Sera were diluted to 1:800, cloacal swab fluid to 1:1, small intestine wash to 1:4 and bile samples to 1:800 in 2.5% skim milk (PBS-T). All the diluted samples were added to marked triplicate wells and incubated at 37 °C for 1 h. Plates were washed three times and IgA or IgG antibodies were detected by adding pre-titrated goat anti-chicken IgA-HRP 1:3000 or IgY-HRP 1:10,000 (Gallus Immunotech, Shirley, MA, USA) diluted in 2.5% skim milk, respectively, and incubated at 37 °C for 1 h. The plates were washed with PBS-T and 3,3′,5,5′-Tetramethylbenzidine (TMB; SeraCare, Milford, MA, USA) was added and incubated at room temperature in the dark for 10–20 min. The reaction was stopped when optimal color was developed in wells by adding 1 M phosphoric acid, and the optical density (OD) values at 450 nm were measured in a microplate reader (Molecular Devices, CA, USA).

### 2.4. Splenocyte Proliferation Assay

The procedure for identifying the SE-specific lymphocyte proliferation index was performed as described previously [14]. Briefly, splenocytes were isolated using the Ficoll-Paque method and were incubated at 39 °C in an atmosphere of 5% CO_2_ for 48 h, together with 10 µg/mL of OMP+FLA. CellTiter 96^®^ Aqueous One Solution Reagent (Promega, Madison, WI, USA) was used to determine the antigen-specific lymphocyte proliferation response by incubation with the cells for 4 h. Optical density (OD) values were recorded at 490 nm absorbance, and the stimulation index (SI) was calculated from OD value of stimulated cells divided by the value of unstimulated control cells.

### 2.5. Total and Recall SE Specific Lymphocyte Subsets Frequencies by Flow Cytometry

The frequency of specific T lymphocyte subsets was analyzed both before and after restimulation with SE antigens as described previously [5,18]. Briefly, freshly isolated splenocytes on the day of necropsy were stimulated with 10 µg/mL of OMP+FLA mixture for 72 h. Protein transport inhibitors Brefeldin A (GolgiPlug; BD Bioscience, San Jose, CA, USA) and Monensin (GolgiStop; BD Bioscience, San Jose, CA, USA) were added for the last 6 h of incubation. Cells were harvested, washed, blocked with 1% normal rabbit serum and surface-immunostained using chicken-lymphocyte-specific antibodies, CD3, CD4, CD8α and TCRγδ or the corresponding isotype control antibody tagged with fluorescein or biotin (Table 2). Cells were fixed with 1% paraformaldehyde, washed, and suspended in FACS buffer. Intracellular IFNγ staining was performed as reported previously [18]. The immunostained cells were acquired using BD FACS Aria II (BD Biosciences, San Jose, CA, USA), and the data were analyzed using the FlowJo software (Tree Star, Ashland, OR, USA). The following antibody panels were used to elucidate the CD8 and CD4 positive T cell subsets.

(i) CD8^+^ T cell panel: mouse anti-chicken CD3 AF700; mouse anti-chicken CD8a PE; mouse anti-chicken TCRγδ biotin; streptavidin AF488; rabbit anti-chicken IFNγ and goat anti-rabbit IgG AF647.

(ii) CD4^+^ T cell panel: mouse anti-chicken CD3 AF700; mouse anti-chicken CD4 FITC; mouse anti-chicken TCRγδ biotin; streptavidin PE.

### 2.6. RNA Isolation and Quantitative Real-Time PCR (qRT-PCR)

The mRNA expression levels of chicken TLRs and cytokines were analyzed as described previously [15]. Briefly, total RNA from the cecal tonsils was extracted using a TRIzol reagent (Molecular Research Center, Cincinnati, OH, USA) as per the manufacturer’s instructions. The purity and concentration of RNA was determined by a NanoDrop 2000 Spectrophotometer (Thermo Scientific, Waltham, MA, USA). The cDNA synthesis was achieved as reported earlier [5] by using 2 ng RNA as a template. The mRNA expression was analyzed and quantified by a 7500 Real-Time PCR System spectrofluorometric thermocycler (Applied Biosystems, Waltham, MA, USA) using PerfeCTa SYBR Green SuperMix (Quantabio, Beverly, MA, USA). The protocol and cycle conditions used in qRT-PCR were reported earlier [5]. Primers and annealing temperatures are listed in Table 3. The specificity of qRT-PCR product was verified through the melting curve generation at the end of each qRT-PCR run. All target gene expressions were normalized with the mRNA level of the housekeeping gene (β-actin) and reported as the fold change (2^–∆∆^Ct method) [19].

### 2.7. Statistical Analysis

Statistical analyses were conducted on data using GraphPad Prism software (GraphPad Software, Inc., La Jolla, CA, USA). Values were reported as mean ± standard error of mean (SEM) from 6–13 birds in each group. One-way analysis of variance (ANOVA) with Tukey’s test was used to determine the statistically significant differences between the groups. Significance was established at *p* < 0.05.

### 2.8. Ethics Statement

In accordance with the recommendations of Public Health Service Policy, the United States Department of Agriculture Regulations, the National Research Council’s Guide for the Care and Use of Laboratory Animals, and the Federation of Animal Science Societies’ Guide for the Care and Use of Agricultural Animals in Agricultural Research and Teaching, the animal study was carried out. We followed all relevant institutional, state, and federal regulations and policies regarding animal care and use at Ohio State University. Broiler birds were maintained, samples were collected, and birds were euthanized in accordance with the approved protocol of the Institutional Animal Care and Use Committee at Ohio State University (Protocol number 2016A00000060-R1).

## 3. Results

### 3.1. Pre-Challenge Antibody Response against Salmonella Antigens

**Figure 1:**
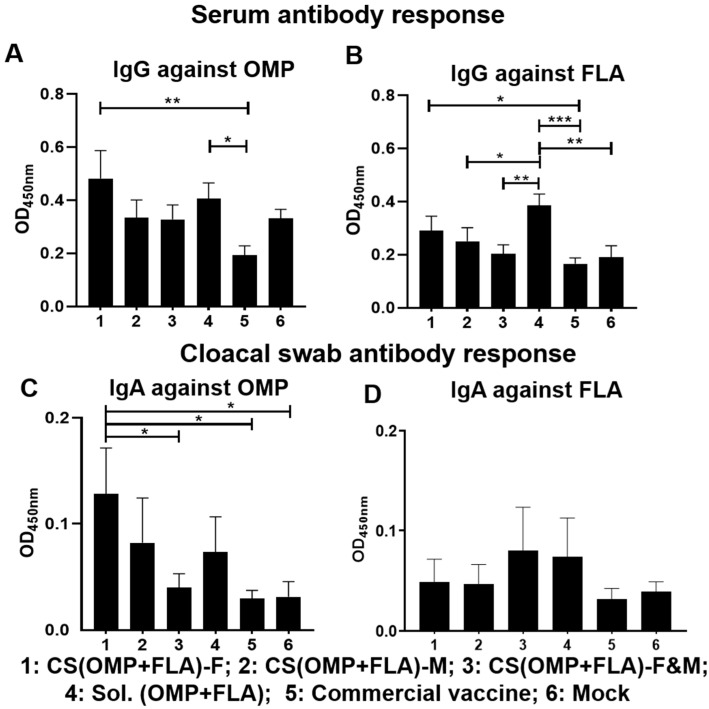
Post-vaccination *Salmonella* antigens’ specific IgG and IgA antibody responses. Broilers were vaccinated with CS(OMP+FLA)-F, CS(OMP+FLA)-M and CS(OMP+FLA)-F&M orally at age 3 days, with each dose containing 10 µg (OMP+FLA), followed by a booster dose at age 3 weeks. The control birds received mock saline, 10 µg of Sol.Ag (OMP+FLA) and commercial live *Salmonella* vaccine orally on the same days of our candidate vaccines schedule. Blood and cloacal swab samples collected at week 5 (2 weeks after booster) post-vaccination were analyzed for detection of specific IgG and IgA antibodies, respectively, by ELISA. Serum samples were analyzed for IgG antibody response against (**A**) OMP and (**B**) FLA protein. Cloacal swabs were analyzed for IgA antibody response against (**C**) OMP and (**D**) FLA protein. Data are presented as the mean ± SEM of 6 to 13 birds per group. Significant differences were determined by one-way ANOVA followed by Tukey post-hoc test between each of the groups. Only significant differences are labelled in the figures (* *p* < 0.05, ** *p* < 0.01, and *** *p* < 0.001).

Broiler chickens were purchased from a commercial hatchery for testing the efficacy of our candidate oral-delivered CS-based *Salmonella* subunit vaccine formulations and compared that with a commercial live vaccine (Poulvac^®^ ST, Kalamazoo, MI, USA) administered orally on the same day. To monitor the vaccine specific antibody levels before challenge, blood and cloacal swab samples were collected after one and 2 two doses of vaccination at ages 3 and 5 weeks, respectively. After the first dose, vaccination serum IgG titers against OMP and FLA were not significantly different among all the experimental groups, and specific IgA response in cloacal swab was very low (OD < 0.05) (data not shown). After the booster dose inoculation, the CS(OMP+FLA)-F vaccinates had significantly (*p* < 0.01 and *p* < 0.05) increased anti-OMP and anti-FLA IgG antibody levels compared to commercial vaccine (Figure 1A,B). The Sol.Ag vaccinates had a significantly (*p* < 0.05) increased anti-OMP IgG antibody level compared to commercial vaccine (Figure 1A), and significantly (*p* < 0.001 to *p* < 0.05) increased IgG antibody response against FLA than commercial, CS(OMP+FLA)-F&M and CS(OMP+FLA)-M vaccinates and mock birds (Figure 1B). The CS(OMP+FLA)-F vaccinates had a significantly (*p* < 0.05) higher anti-OMP IgA antibody level in cloacal swab compared to commercial and CS(OMP+FLA)-F&M vaccines and the mock group of birds, while CS(OMP+FLA)-F&M vaccinates had lesser antibody levels compared to Sol.Ag vaccinates (Figure 1C). Although CS(OMP+FLA)-F&M and Sol.Ag vaccinates had higher anti-FLA IgA antibody levels in cloacal swab compared to all the other groups, the data was not statistically significant (Figure 1D).

### 3.2. Post-Challenge Antibody Response against Salmonella Antigens

**Figure 2:**
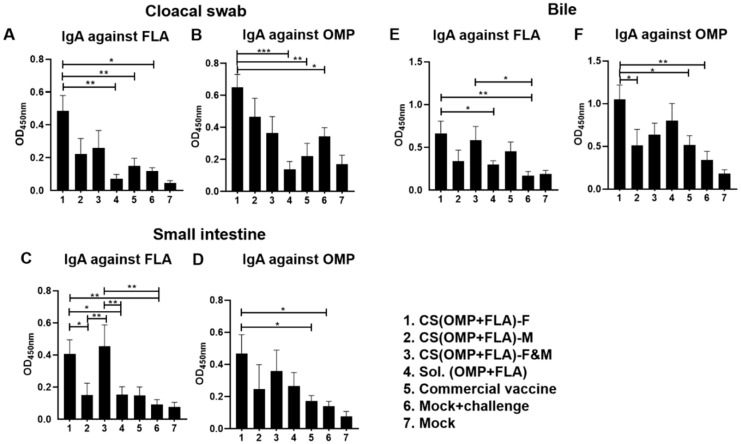
*Salmonella* antigen-specific IgA antibody in vaccinated and infected birds. Broilers were vaccinated as described in figure legend 1, challenged at the age of 5 weeks with *Salmonella* Enteritidis, and euthanized 10 days post-challenge, and samples were collected for specific IgA antibody analysis by ELISA. The anti-FLA IgA antibody response in (**A**) cloacal swab, (**C**) small intestine wash, and (**E**) bile samples. The anti-OMP IgA antibody response in (**B**) cloacal swab, (**D**) small intestine wash, and (**F**) bile samples. Data are presented as the mean ± SEM of 6 to 13 birds per group. Significant differences were determined by one-way ANOVA followed by Tukey post-hoc test between each of the groups. Only significant differences are labelled in the figures (* *p* < 0.05, ** *p* < 0.01, and *** *p* < 0.001).

*Salmonella* antigen-specific serum IgG antibody levels were not significantly different in all the experimental groups after challenge infection (data not shown). In contrast, experimental vaccines enhanced the antigen-specific IgA antibody levels in post-challenge samples (Figure 2). The CS(OMP+FLA)-F vaccination increased (*p* < 0.001 to *p* < 0.05) both anti-FLA and anti-OMP IgA antibody levels in cloacal swab over the control groups (Figure 2A,B). Notably, CS(OMP+FLA)-F&M and CS(OMP+FLA)-F vaccinates had significantly (*p* < 0.01 and *p* < 0.05) increased IgA antibody levels specific to FLA antigens in the small intestinal wash compared to the CS(OMP+FLA)-M and Sol.Ag vaccine and mock-challenge groups (Figure 2C). OMP-specific IgA antibody response in small intestinal wash was significantly (*p* < 0.01 and *p* < 0.05) increased in CS(OMP+FLA)-F vaccinates compared to commercial vaccine and mock-challenge birds (Figure 2D). CS(OMP+FLA)-F vaccination significantly (*p* < 0.01 and *p* < 0.05) enhanced anti-FLA IgA antibody levels in the bile compared to both Sol.Ag and mock-challenge groups, and CS(OMP+FLA)-F&M vaccination increased (*p* < 0.05) that response over mock-challenge groups (Figure 2E). Anti-OMP IgA antibody levels in bile was increased by CS(OMP+FLA)-F vaccination over other groups, and the level was significantly (*p* < 0.01 and *p* < 0.05) higher compared to the CS(OMP+FLA)-M, commercial vaccine and mock-challenge groups of birds (Figure 2F).

### 3.3. Live Vaccine and Challenge Bacterial Load in Vaccinated Birds

The cloacal swab fluid of birds after the first and second dose of commercial live *Salmonella* vaccination was pooled by group and plated on XLD and BGA plates to monitor *Salmonella* load before challenge infection. Except birds that received the commercial vaccine, all other groups remained negative for *Salmonella,* and vaccine bacteria were detectable after 14 days from the first vaccination and negative after the second vaccination before SE challenge infection at age 5 weeks.

**Figure 3:**
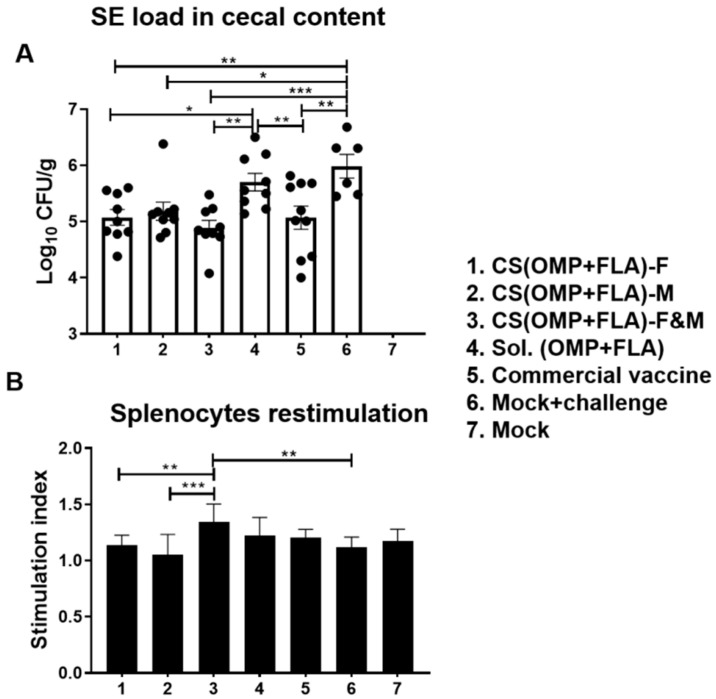
*Salmonella* Enteritidis load in the cecal content and antigen-specific lymphocyte proliferation response in splenocytes of vaccinated and challenged birds. (**A**) *Salmonella* CFU was enumerated in cecal content by plating on XLD agar plates and theindividual bird values were dot-plotted. (**B**) Splenocytes harvested on the day of necropsy were stimulated with 10 µg/mL SE antigens (OMP+FLA) for 48 h and lymphocyte stimulation index value was calculated by the mean OD of specific antigen (OMP+FLA) stimulated proliferation/mean OD of non-stimulated proliferation by a colorimetric assay. Data are presented as the mean ± SEM of 6 to 13 birds per group. Significant differences were determined by one-way ANOVA followed by Tukey post-hoc test between each of the groups. Only significant differences are labelled in the figures (* *p* < 0.05, ** *p* < 0.01, and *** *p* < 0.001).

The cecal content of birds was collected on the day of necropsy, and CFUs were enumerated on XLD agar plates. The SE CFU was undetectable in mock group birds (Figure 3A). Among the vaccinated groups, birds that received CS(OMP+FLA)-F (log_10_ 5.08 CFU/g), CS(OMP+FLA)-M (log_10_ 5.18 CFU/g), CS(OMP+FLA)-F&M (log_10_ 4.89 CFU/g), and commercial vaccine (log_10_ 5.07 CFU/g) had a significantly (*p* < 0.001 to *p* < 0.05) reduced challenge SE load compared to mock-challenge birds (log_10_ 5.99 CFU/g) (Figure 3A). Besides, CS(OMP+FLA)-F&M, CS(OMP+FLA)-F, and commercial vaccines had significantly (*p* < 0.01 and *p* < 0.05) reduced bacterial load compared to the Sol.Ag vaccine group (Figure 3A). Although, among those birds that received the three CS(OMP+FLA)-based and commercial live *Salmonella* vaccines, the data on SE reduction was not statistically significant, birds that received CS(OMP+FLA)-F+M (log_10_ 4.89 CFU/g) had the lowest (*p* < 0.001) SE load reduction, of over one log_10_ CFU/g of cecal content, compared to the mock-challenge group (Figure 3A).

### 3.4. Salmonella Antigens (OMP+FLA) Specific Recall Lymphocyte Proliferation Response in Vaccinates

In splenocytes of CS(OMP+FLA)-F&M vaccine-receiving birds, an increased trend in OMP and FLA antigen-specific lymphocyte proliferation stimulation index was detected, compared to all the other experimental groups, while that increased response was significantly (*p* < 0.001 and *p* < 0.01) higher compared to CS(OMP+FLA)-M and CS(OMP+FLA)-F vaccines and in the mock-challenge group (Figure 3B).

### 3.5. Both the Total and Antigen Specific Recall T Cell Subset Frequencies in CS(OMP+FLA) Vaccinates

The antigen-specific T cell frequencies were detected based on the expression of a combination of surface [CD8^+^ T cell and CD4^+^ T cell panels] and intracellular immune markers by using the standard lymphocyte gating strategy in both antigens stimulated (Figure S1) and unstimulated splenocytes (Figure S2) by flow cytometry.

**Figure 4:**
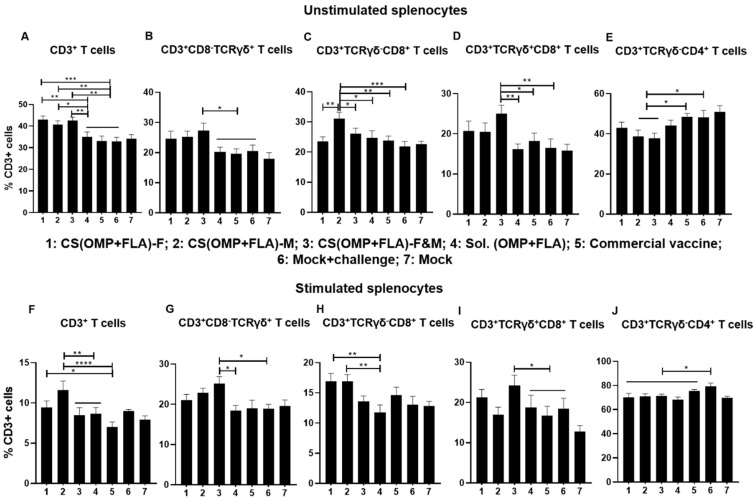
Recall frequencies of various lymphocyte subsets in the spleens of vaccinated and challenged broilers. Splenocytes isolated on the day of necropsy were analyzed for the frequencies of unstimulated and SE (OMP+FLA) antigen-stimulated lymphocyte subsets by flow cytometry. The frequency of (**A**,**F**) CD3^+^ T cells; (**B**,**G**) CD3^+^CD8^-^TCRγδ^+^ T cells; (**C**,**H**) CD3^+^TCRγδ^-^CD8^+^ T cells; (**D**,**I**) CD3^+^CD8^+^TCRγδ^+^ T cells; and (**E**,**J**) CD3^+^TCRγδ^-^CD4^+^ T cells. Data are presented as the average percentage of indicated splenocyte subset ± SEM of 6 to 13 birds per group. Significant differences were determined by one-way ANOVA followed by Tukey post-hoc test between each of the groups. Only significant differences are labelled in the figures (* *p* < 0.05, ** *p* < 0.01, *** *p* < 0.001, and **** *p* < 0.0001).

In unstimulated splenocytes of birds that received CS(OMP+FLA)-based vaccines, significantly higher (*p* < 0.001 to *p* < 0.05) total CD3^+^ T cell frequencies were observed (Figure 4A). In contrast, CS(OMP+FLA)-F&M vaccine significantly (*p* < 0.01 and *p* < 0.05) increased CD3^+^CD8^-^TCRγδ^+^ and γδ T cells (CD3^+^TCRγδ^+^CD8^+^) frequencies compared to in the commercial vaccine, Sol.Ag and mock-challenge groups (Figure 4B,D). A significantly (*p* < 0.001 to *p* < 0.05) increased frequency of cytotoxic T lymphocytes (CTLs) (CD3^+^TCRγδ^-^CD8^+^) was observed in CS(OMP+FLA)-M vaccinates compared to in the remaining groups (except mock) (Figure 4C). The increased frequency of T-helper cells (CD3^+^TCRγδ^-^CD4^+^) was significantly (*p* < 0.05) higher in the commercial vaccine and mock-challenge groups compared to the CS(OMP+FLA)-M and F&M groups (Figure 4E).

In *Salmonella* antigen (OMP+FLA) stimulated splenocytes, the CS(OMP+FLA)-M vaccine significantly (*p* < 0.0001 and *p* < 0.01) increased the total CD3^+^ T cells compared to commercial, CS(OMP+FLA)-F&M and Sol.Ag vaccines (Figure 4F). The CS(OMP+FLA)-F vaccine increased total CD3^+^ T cell frequency, which was significantly (*p* < 0.05) higher compared to the commercial vaccine group (Figure 4F). Like in unstimulated cells, in antigen-stimulated cells, the CS(OMP+FLA)-F&M vaccine significantly (*p* < 0.05) upregulated CD3^+^CD8^-^TCRγδ^+^ and γδ T cell frequency compared to Sol.Ag and mock-challenge groups (Figure 4G,I). Both CS (OMP+FLA)-M and CS (OMP+FLA)-F vaccines had a significantly (*p* < 0.01) increased frequency of CTLs compared to the Sol.Ag vaccine group (Figure 4H). The T-helper cell frequency was significantly (*p* < 0.05) increased in the mock-challenge group compared to the Sol.Ag, the commercial and all the CS(OMP+FLA)-based vaccine groups (Figure 4J). We observed increased IFNγ-secreting CTLs with the CS(OMP+FLA)-F vaccine, while other cell frequencies were not significantly altered by the CS(OMP+FLA)-based vaccines (date not shown).

### 3.6. CS(OMP+FLA) Vaccine-Induced TLRs and Cytokine mRNA Expression in Cecal Tonsils

**Figure 5:**
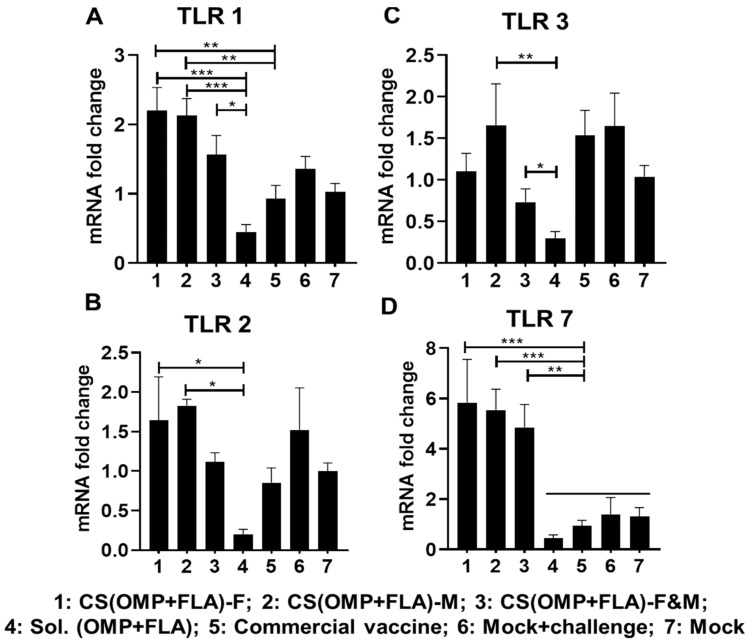
Toll-like receptors (TLRs) gene expression in vaccinated and challenged birds. Cecal tonsils collected on the day of necropsy were analyzed for the expression of TLR mRNA by qRT-PCR. The fold changes of (**A**) TLR 1, (**B**) TLR 2, (**C**) TLR 3, and (**D**) TLR 7. Fold change in gene expression was calculated after correcting for β-actin mRNA value and normalizing to mRNA content of mock group as 1. Data are presented as the mean ± SEM of 6 to 13 birds per group. Significant differences were determined by one-way ANOVA followed by Tukey post-hoc test between each of the groups. Only significant differences are labelled in the figures (* *p* < 0.05, ** *p* < 0.01, and *** *p* < 0.001).

All the analyzed TLRs gene expressions were higher with the CS (OMP+FLA)-F and CS (OMP+FLA)-M vaccines compared to in the CS (OMP+FLA)-F&M vaccine group (Figure 5). The CS(OMP+FLA)-based-vaccine-receiving birds expressed a significantly (*p* < 0.001 to *p* < 0.05) higher TLR 1 mRNA level compared to the Sol.Ag vaccine group (Figure 5A). Especially, CS(OMP+FLA)-F and CS(OMP+FLA)-M vaccinates had a statistically (*p* < 0.01) higher expression of TLR 1 compared to the commercial vaccine group (Figure 5A), whereas these vaccines increased TLR 2 expression more (*p* < 0.01 and *p* < 0.05) than in the Sol.Ag vaccine group (Figure 5B). The CS(OMP+FLA)-M vaccine significantly (*p* < 0.01) increased TLR 3 gene expression compared to the Sol.Ag group, while both the mock-challenge and commercial vaccine groups had a comparable increased level of TLR3 expression (Figure 5C). The CS(OMP+FLA)-F&M vaccine group had a significantly increased (*p* < 0.05) TLR 3 gene expression compared to the Sol.Ag vaccine group (Figure 5C). The CS(OMP+FLA)-based vaccines significantly (*p* < 0.001 and *p* < 0.01) enhanced TLR 7 gene expression compared to all the control groups (Figure 5D).

**Figure 6:**
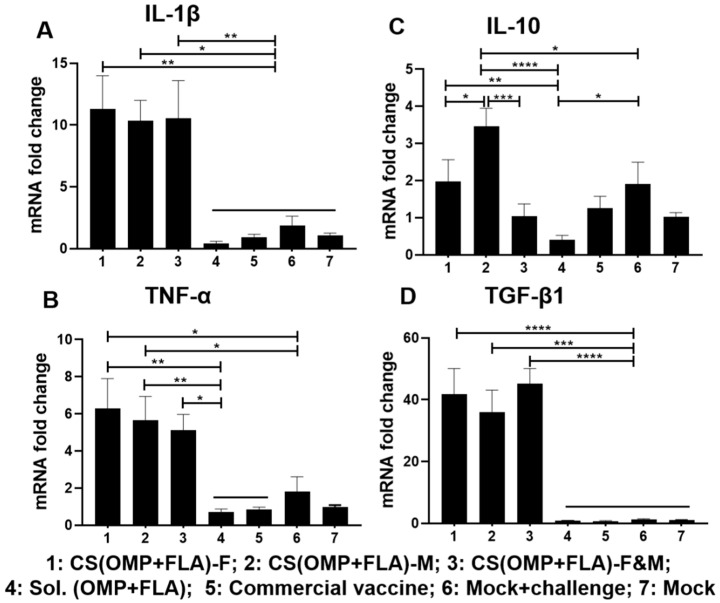
Cytokine gene expression in vaccinated and challenged birds. Cecal tonsils collected on the day of necropsy were analyzed for the expression of different cytokine mRNA levels by qRT-PCR. The gene expression of (**A**) IL-1β, (**B**) TNF-α, (**C**) IL-10, and (**D**) TGF-β1. Fold change in gene expression was calculated after correcting for β-actin mRNA value and normalizing to the mRNA content of the mock group as 1. Data are presented as the mean ± SEM of 6 to 13 birds per group. Significant differences were determined by one-way ANOVA followed by Tukey post-hoc test between each of the groups. Only significant differences are labelled in the figures (* *p* < 0.05, ** *p* < 0.01, *** *p* < 0.001, and **** *p* < 0.0001).

The CS(OMP+FLA)-based vaccines significantly (*p* < 0.0001 to *p* < 0.05) increased IL-1β and TGF-β1 mRNA expression compared to all the other experimental groups (Figure 6A,D), while the increased TNF-α expression by these vaccines was higher (*p* < 0.01 and *p* < 0.05) than in the Sol.Ag and commercial vaccine groups (Figure 6B). In contrast, both (OMP+FLA)-F and CS (OMP+FLA)-M vaccine groups had a significantly (*p* < 0.05) increased expression of TNF-α compared to the mock-challenge group (Figure 6B). The CS (OMP+FLA)-M vaccine group had significantly increased (*p* < 0.0001, *p* < 0.001, and *p* < 0.05) IL-10 gene expression relative to the Sol.Ag, CS (OMP+FLA)-F&M, CS(OMP+FLA)-F and mock-challenge groups (Figure 6C). The (OMP+FLA)-F vaccine and mock-challenge groups had a significantly (*p* < 0.01 and *p* < 0.05) increased expression of IL-10 compared to the Sol.Ag vaccine group (Figure 6C).

## 4. Discussion

Vaccination is a widely adopted approach to control SE colonization in poultry [20]. To achieve maximum protection against *Salmonella* by any candidate vaccines, it is important to understand the immune mechanism. By using two doses of 10 μg antigens in each dose of CS(OMP+FLA)-F, -M and F&M vaccine formulation, a reduction in SE colonization in cecum was achieved which was comparable to commercial live vaccine. Among the three CS(OMP+FLA)-based formulations, the maximum bacterial reduction, 1.1 log_10_ CFU/g, was observed in birds that received CS(OMP+FLA)-F&M vaccine. Higher secretion of mucosal antigen-specific IgA responses was detected in CS(OMP+FLA)-F vaccinates, followed by CS(OMP+FLA)-F&M vaccinates, while both the vaccines did not increase the IgG antibody response. The gastrointestinal tract is the predilection site for colonization of *Salmonella*, and it augments local mucosal IgA secretion and prevents bacterial attachment to the epithelial cells [21]. In our previous studies in layers and broilers, CS(OMP+FLA)-F vaccine oral inoculation elicited robust specific local IgA antibody rather than IgG antibody responses and provided partial protection against *Salmonella* colonization [5,6,15]. A study in mice showed that mannosylated chitosan microsphere-based vaccine delivered orally binds to mannose receptors on macrophages and triggers the IgA antibody response [9]. Like our previous studies, in this study, we also confirmed that CS(OMP+FLA)-F vaccine enhanced the local mucosal antibody responses, which translated into protection [5,6].

In chicken, several studies have shown the critical role of cell-mediated immunity in providing protection against *Salmonella* [22,23]. In the present study, CS(OMP+FLA)-F&M vaccine induced higher CD3^+^, CD3^+^CD8^-^TCRγδ^+^ and γδ T cell responses, whereas CS(OMP+FLA)-M vaccine enhanced CTL generation. In an earlier experiment, the oral vaccination induced robust γδ T cell populations, which played an important role in providing protection of chickens against *Salmonella* colonization. γδ T cells were suggested to facilitate mucosal defense against intestinal pathogens in chickens [24]. In mice, the presence of γδ T cells was important to confer resistance to *Salmonella* infection [25,26]. Mannose ligand binds mannose receptor and mediates the antigen internalization by DCs, leading to its presentation to naive T cells [27]. Mannosylated antigen enhances the Th1 immune responses through MHC class I antigen presentation to CD8^+^ T cells [28]. Mannosylated nanoparticle-based vaccine stimulates CTLs and enhances Th1-based immunity [29]. Mannan-ligand-coated nanoparticles enhance CD4^+^ and CD8^+^ T-cell responses compared to uncoated nanoparticles [30]. Together, the increased cell-mediated immunity induced by mannose-based CS(OMP+FLA)-M and CS(OMP+FLA)-F&M vaccines correlated with slightly improved efficiency in clearance of *Salmonella*, an intracellular bacterium.

The expression of several TLRs (1, 2, 3 and 7) and cytokines (IL-1β, TNF-α, IL-10 and TGF-β1) mRNA was observed in cecal tonsils of CS(OMP+FLA)-based-vaccine-inoculated birds. Increased expression of IL-1β and TNF-α indicated a proinflammatory response, and their inflammatory effect was balanced by the anti-inflammatory cytokines IL-10 and TGF-β1. Similarly, in our earlier experiments, CS(OMP+FLA)-F vaccine enhanced various TLRs and Th1 and Th2 cytokine gene expression [5,6,15]. An effective defense against *Salmonella* requires induction of both innate and adaptive immune responses [31,32]. TLRs are associated with the activation of innate immune molecules, which leads to activation of adaptive immune responses [33]. TLR -1 and TLR -2 were shown to cooperate in recognizing bacterial lipoprotein, restriction of bacterial movement and activation of proinflammatory cytokine production [33,34,35]. Additionally, chicken TLR 7 was shown to increase IL-1β secretion [36], which is consistent with elevated IL-1β mRNA expression in the cecal tonsils of vaccinated birds in our study. IL-1β is involved in protection against *Salmonella* infection by limiting its entry into systemic sites [37]. Besides, mannose as an adjuvant, when conjugated on nanoparticles, targets mannose receptors on dendritic cells and helps in their activation via the TLR-dependent signaling pathway [38,39]. The TLR ligands synergize with mannose receptors, leading to bridging of innate and adaptive immune responses [40].

In the present study, commercial-live-vaccine-induced SE load reduction was comparable to that of CS(OMP+FLA)-based vaccines. CS(OMP+FLA)-based vaccines induced higher antibody and/or cellular immune responses, correlated with improved protective efficiency, but the commercial vaccine did not induce any such substantial immune response, suggesting the need for further studies to understand its mechanism of induction of protection in broilers. However, the commercial live vaccine stimulated significant levels of specific IgG and IgA responses in layer birds [41]. This may be due to the age difference between broilers and layers at the time of vaccination. The commercial vaccine failed to induce any pro- or anti-inflammatory cytokine production associated with poor T-cell responses in broilers [42].

## 5. Conclusions

The mannose-conjugated CS(OMP+FLA)-F&M vaccine enhanced both cellular and antibody responses, whereas the CS(OMP+FLA)-F vaccine increased more mucosal antibody responses. However, both the FLA and mannose-conjugated CS vaccine formulations induced TLRs and balanced Th1 and Th2 cytokine gene expression and reduced the *Salmonella* load in the intestines of broilers. The present study confirmed the earlier rodent studies that found that mannose delivered in nanoparticle vaccine formulations improve the level of protection against *Salmonella*.

## Figures and Tables

**Table 1:** Experimental animal groups.

Group	Vaccine Received	N	1st Dose/Age	2nd Dose/Age	Challenge/Age
1	10 µg CS(OMP+FLA)-F	9	3 day	3 week	5 week
2	10 µg CS(OMP+FLA)-M	9	3 day	3 week	5 week
3	10 µg CS(OMP+FLA)-F&M	9	3 day	3 week	5 week
4	10 µg Sol.Ag (OMP+FLA)	9	3 day	3 week	5 week
5	Commercial vaccine	10	3 day	3 week	5 week
6	PBS (Mock-challenge)	6	3 day	3 week	5 week
7	PBS (Mock)	13	3 day	3 week	NA

NA: not applicable; N: number of animals used in each group.

**Table 2:** Antibodies used for lymphocyte cell surface and intracellular IFNγ immunostaining.

Antibody	Catalog Number and Company Name
Mouse anti-chicken CD3 AF700	Cat# 8200-27; SouthernBiotech, Birmingham, AL, USA
Mouse anti-chicken CD4 FITC	Cat# 8210-02; SouthernBiotech, Birmingham, AL, USA
Mouse anti-chicken CD8α PE	Cat# 8220-09; SouthernBiotech, Birmingham, AL, USA
Mouse anti-chicken TCRγδ biotin	Cat# 8230-08; SouthernBiotech, Birmingham, AL, USA
Streptavidin PE	Cat# 557598; BD Pharmingen, San Jose, CA, USA
Streptavidin AF488	Cat#405235, BioLegend, San Diego, CA, USA
Rabbit anti-chicken IFNγ	Cat# AHP945Z; Bio-Rad; Hercules, CA, USA
Goat anti-rabbit IgG AF647	Cat# 4050-31; SouthernBiotech, Birmingham, AL, USA

**Table 3:** Primers used in the qRT-PCR for quantification of TLRs and cytokine mRNA expression.

Primers	Oligonucleotides (5′–3′)	Annealing Temperature
β-actin	Forward: ACCGGACTATTACCAACACC	56 °C
	Reverse: GACTGCTGCTGACACCTTCA	
TLR 1	Forward: GCTGTGTCAGCATCAGAGGA	58 °C
	Reverse: GTGGTACCTCGCAGGGATAA	
TLR 2	Forward: GCTCAACAGCTTCTCCAAGG	57 °C
	Reverse: CCACCAGGATGAGGATGAAC	
TLR 3	Forward: CCTCCTTGGGACACCTGAAA	54 °C
	Reverse: ATTCCGCAGTGGATGAAAAG	
TLR 7	Forward: AGAGACTGGCTTCCAGGACA	58 °C
	Reverse: CAGCTGAACATACCGGGACT	
IL-1β	Forward: TGGGCATCAAGGGCTACA	57 °C
	Reverse: TCGGGTTGGTTGGTGATG	
IL-10	Forward: CATGCTGCTGGGCCTGAA	57 °C
	Reverse: CGTCTCCTTGATCTGCTTGATG	
TNF-α	Forward: ATCCTCACCCCTACCCTGTC	56 °C
	Reverse: GGCGGTCATAGAACAGCACT	
TGF-β1	Forward: AGGATCTGCAGTGGAGTGGAT	54 °C
	Reverse: CCCCGGGTTGTGTTGGT	

## Supplementary Materials

The following are available online at https://www.mdpi.com/2076-393X/8/2/299/s1, Figure S1. Illustration of the stimulated splenocytes gating strategy. The splenocytes were immunostained by surface and intracellular lymphocyte and cytokine specific antibodies and analyzed by flow cytometry. The flow cytometry gating strategy of (A) Isotype control for CD8^+^ T cell panel; (B) Specific antibody staining for CD8^+^ T cell panel; (C) Isotype control for CD4^+^ T cell panel; and (D) Specific antibody staining for CD4^+^ T cell panel, Figure S2. Illustration of the unstimulated splenocytes gating strategy. The splenocytes were immunostained by surface and intracellular lymphocyte and cytokine specific antibodies and analyzed by flow cytometry. The immune cells gating strategy of (A) Isotype control for CD8^+^ T cell panel; (B) Specific antibody staining for CD8^+^ T cell panel; (C) Isotype control for CD4^+^ T cell panel; and (D) Specific antibody staining for CD4^+^ T cell panel.
