# Supplementary material for: Mannose-Modified Chitosan-Nanoparticle-Based Salmonella Subunit OralVaccine-Induced Immune Response and Efficacy in a Challenge Trial in Broilers
Source: Vaccines (Basel). 2020 Jun 11;8(2):299. doi: 10.3390/vaccines8020299 (PMC7349978; doi:10.3390/vaccines8020299)
Supplement: Supplementary file 1 [file vaccines-08-00299-s001.pdf]

vaccines

MDPI

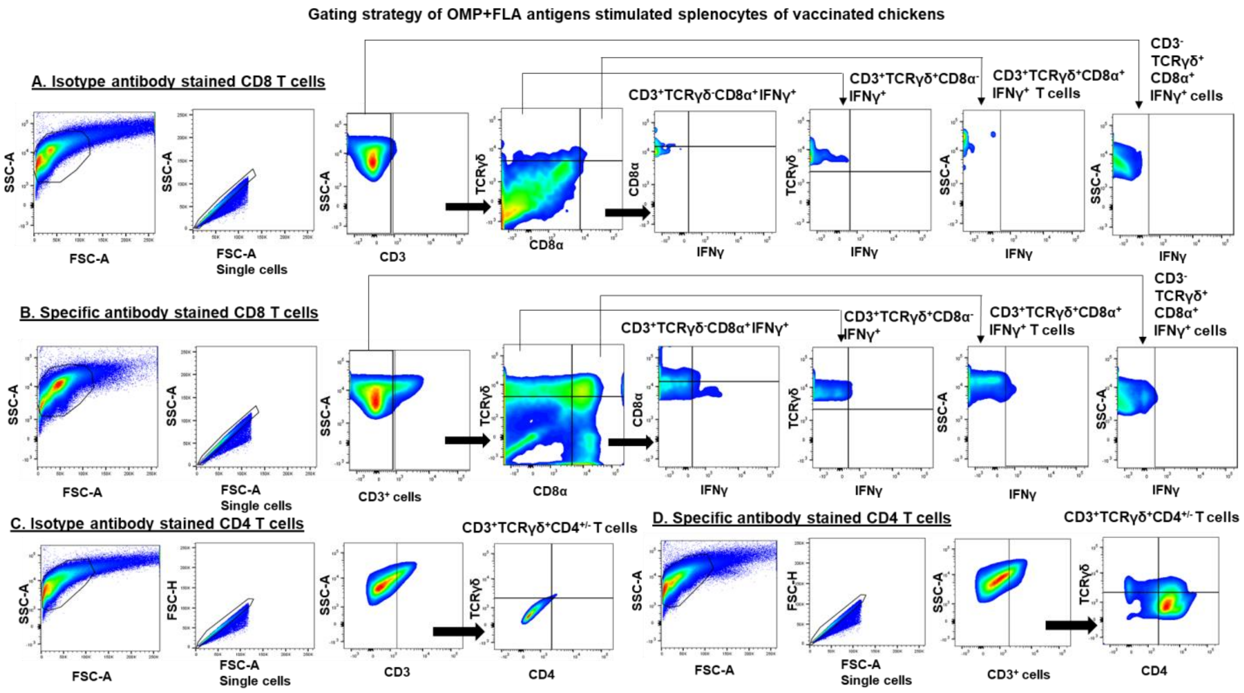

Figure S1. Illustration of the stimulated splenocytes gating strategy. The splenocytes were immunostained by surface and intracellular lymphocyte and cytokine specific antibodies and~~were~~ analyzed by flow cytometry. The flow cytometry gating strategy of (A) Isotype control for $CD8^{+}$ T cell panel; (B) Specific antibody staining for $CD8^{+}$ T cell panel; (C) Isotype control for $CD4^{+}$ T cell panel; and (D) Specific antibody staining ~~control~~ for $CD4^{+}$ T cell panel.

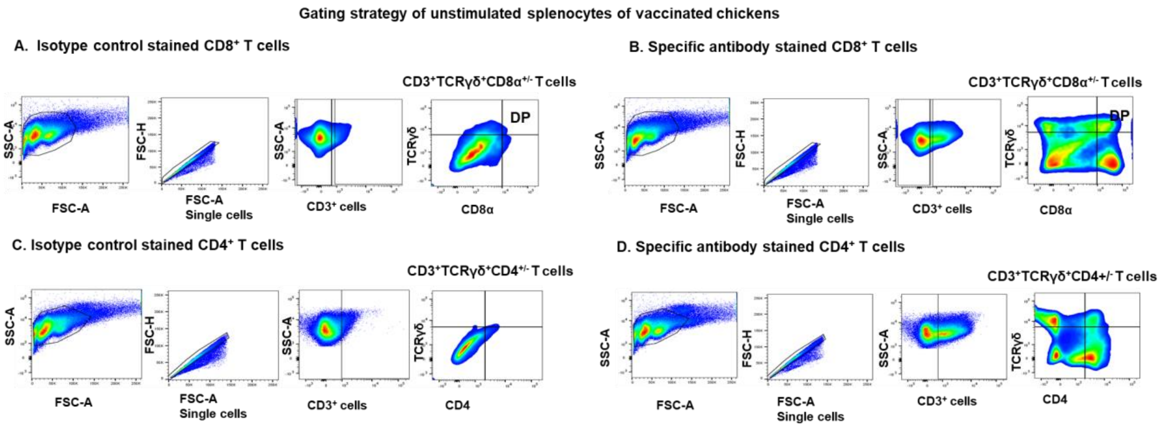

Figure S2. Illustration of the unstimulated splenocytes gating strategy. The splenocytes were immunostained by surface and intracellular lymphocyte and cytokine specific antibodies and analyzed by flow cytometry. The immune cells gating strategy of (A) Isotype control for CD8$^+$ T cell panel; (B) Specific antibody staining ~~control~~ for CD8$^+$ T cell panel; (C) Isotype control for CD4$^+$ T cell panel; and (D) Specific antibody staining ~~control~~ for CD4$^+$ T cell panel.
